# Co‐Designing a Multimodal Physical Activity Intervention for Individuals With Young‐Onset Type 2 Diabetes (18–40 Years) in China

**DOI:** 10.1111/hex.70580

**Published:** 2026-02-03

**Authors:** Xiaoyan Zhao, Angus Forbes, Haya Abu Ghazaleh, Li Cheng, Xiaodi Guo, Maria Duaso

**Affiliations:** ^1^ Division of Care for Long Term Conditions, Florence Nightingale Faculty of Nursing, Midwifery & Palliative Care, King's College London London UK; ^2^ School of Nursing, Faculty of Medicine, Sun Yat‐sen University Guangzhou China; ^3^ Department of Endocrinology and Metabolism The Third Affiliated Hospital, Sun Yat‐Sen University Guangzhou China

**Keywords:** co‐design, design thinking, intervention development, physical activity, type 2 diabetes, young adults

## Abstract

**Background:**

A limited number of physical activity programmes exist for Chinese people with young‐onset (18–40 years) type 2 diabetes amid its rising global prevalence. This study aims to develop a multimodal intervention for improving physical activity levels for individuals with young‐onset type 2 diabetes using co‐design.

**Methods:**

The development process included three stages. Stage 1 involved synthesising the findings of a review of existing physical activity interventions and a qualitative study of exercise experiences of young adults with type 2 diabetes. This generated a list of candidate intervention elements and behaviour change techniques to inform the co‐design process. Stage 2 involved the development of animated trigger films, using findings from stage 1, to present the physical activity experiences of people with young‐onset type 2 diabetes. In stage 3, a series of co‐design workshops engaging relevant stakeholders were conducted, utilising the outputs from the previous two stages and aligning with the Design Thinking theory.

**Results:**

Twenty‐five participants (12 young adults with type 2 diabetes, 12 healthcare professionals, and one family member) attended co‐design workshops to develop the intervention. The co‐design process resulted in a logic model for a tailored programme–IPAYD (Improving Physical Activity in people with Young‐onset type 2 Diabetes). This programme integrates behaviour change techniques across four elements: individualised goal setting and planning, exercise monitoring, a peer support forum, and educational resources. An eHealth platform was preferred to deliver the programme, incorporating one‐to‐one consultations and optional group sessions to enhance social support and social interaction.

**Conclusions:**

Through stakeholder engagement in a co‐design process, this study makes a novel and much‐needed contribution to developing a physical activity intervention for Chinese people with young‐onset type 2 diabetes.

**Patient and Public Contribution:**

An advisory group of six Chinese young people with type 2 diabetes met online and communicated through a project‐focused WeChat group. They contributed to the animated film scripts, the topic guide of the workshops, the design of the intervention materials, and how to conduct the workshops to align with Chinese culture.

## 1 Introduction

Diabetes affects 589 million adults globally (7.2% of the population) in 2024, with rising prevalence among young adults [1]. In 2013, 2.9% (63 million) of type 2 diagnoses occurred in those aged 20–39 years, and this increased to 3.8% (260 million) in 2021 [1]. A recent national survey in China reported a prevalence of diabetes of 2.0% among individuals aged 18–29 years and 6.3% among those aged 30–39 years [2]. This continued increase is related to the consumption of high‐energy foods and reduced exercise levels, with corresponding increases in obesity [3, 4]. In response to the substantial burden from chronic diseases, the Chinese government has made substantial investments, increasing funding by ¥100–150bn (£10.64–15.95bn) [5]. However, young‐onset type 2 diabetes poses unique challenges, as individuals living with this condition are often in education or employment. The associated psychological strain and reduced quality of life can lead to productivity losses and broader societal impact, particularly in middle‐ and high‐income countries [6, 7]. Evidence is also accumulating that the onset of diabetes at a younger age is associated with greater years of life lost, more rapid β‐cell decline, higher risk of complications, and higher rates of premature mortality and morbidity [3, 7].

Physical activity is a cornerstone of diabetes management, increasing insulin sensitivity, reducing diabetes complications, and improving overall wellbeing [8]. Diabetes control has been prioritised in the Healthy China 2030 initiative, emphasising healthy lifestyle including physical activity should be the foundation, and individuals are the first responsibility for their health [9]. Therefore, increasing engagement in physical activity is important for people with type 2 diabetes. Reported behavioural approaches to improve physical activity in this population include structured exercise programmes, educational and psychological interventions [10]. It is also important to improve the accessibility of exercise facilities, education, and workplace wellness initiatives [11, 12]. However, a large proportion of people with type 2 diabetes do not achieve recommended physical activity levels [13]. People with young‐onset type 2 diabetes face unique challenges in engagement with physical activity, including lack of time due to family, education or employment commitments. Obesity and diabetes‐related stigma may further hinder their participation in exercise [7]. Currently, there is no evidence‐based physical activity programme for Chinese people with young‐onset type 2 diabetes.

As a person‐centred participatory research method where patients and staff work in partnership to develop interventions or services, through adoption of design tools and ways of thinking, co‐design has drawn growing attention in the last decade [14, 15]. Although co‐design shares similar principles and mechanisms with co‐production and co‐creation, and these terms are often used interchangeably, co‐design places greater emphasis on harnessing the contextualised experiences and expertise of related stakeholders through an inclusive and collaborative process of developing intervention ideas [15, 16]. Co‐design has been applied to the development of complex interventions across a range of health‐related behaviours, and its principles are increasingly central to the landscape of care in long term conditions [17, 18]. However, the co‐design process is still new to the intervention design for young‐onset type 2 diabetes and has not yet been integrated into developing physical activity programmes for this population [10]. This study involved people with diabetes and healthcare professionals in co‐designing a programme to increase physical activity that reflects the needs and challenges of young adults with type 2 diabetes.

## 2 Methods

This study used a co‐design methodology called Design Thinking [19]. Design Thinking is comprised of three interconnected phases:

- Inspiration: Identifying the physical, social, and emotional needs of the target groups and the barriers to meeting those needs.
- Ideation: In this phase, participants consider the issues raised in the inspiration phase to identify and prioritise potential solutions to meet these needs.
- Implementation: The intervention prototype developed in the ideation phase is evaluated.

**Figure 1:**
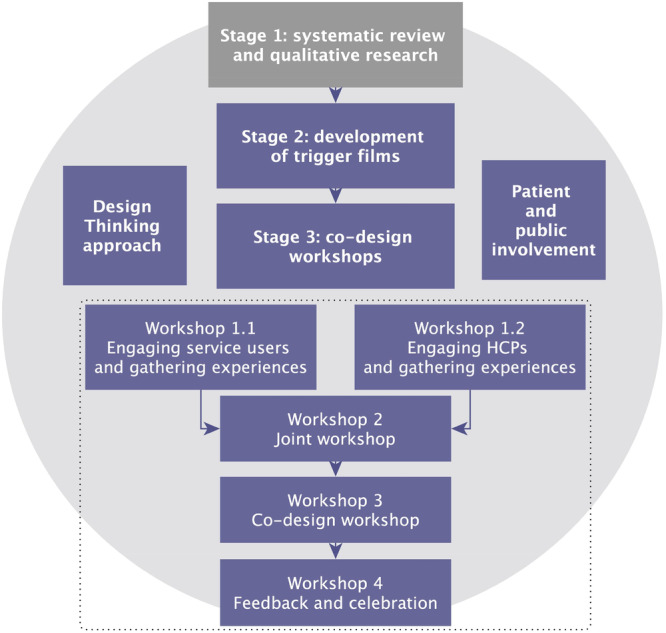
Evidence and theory‐based approach for the intervention development process.

In this paper, we present the intervention development process following the inspiration and ideation phases. Implementation will be addressed in a future feasibility study. This study follows the Medical Research Council's framework for intervention development. It therefore integrates insights (knowledge, theory, and experiences) from a review of physical activity interventions for people with diabetes and a qualitative study detailing the views and experiences of physical activity of young people with diabetes. An overview of the intervention development process is shown in Figure 1.

### 2.1 Stage 1: Evidence From Systematic Review and Qualitative Research

In Stage 1 of the process, we conducted: a systematic review and meta‐analysis of the existing behavioural interventions for improving physical activity in working‐age people with type 2 diabetes; and a qualitative study to explore the barriers and facilitators to physical activity in young‐onset type 2 diabetes [10, 20, 21].

The systematic review, using quantitative evidence synthesis including meta‐analysis and subgroup analysis, demonstrated that psychologically modelled education incorporating behaviour change techniques (BCTs) may be the most effective way to promote physical activity and glycaemic control [10, 20]. From this, a set of effective BCTs was identified, including self‐monitoring, goal setting, action planning, and social support. Findings from the qualitative study confirmed and validated the results of the meta‐analysis [21]. For example, physical activity knowledge and self‐monitoring were identified as psychological capabilities that facilitate physical activity engagement, while guidance from professionals and emotional support represented social opportunities that promote physical activity. These findings informed a list of candidate intervention components and BCTs (see Supporting Information: Material 1) for use in the prioritisation exercise in Stage 3.

The methodological triangulation involved in this stage, combining quantitative and qualitative research methods, helped not only generate a comprehensive list of candidate intervention components and BCTs but also strengthen the validity of the results, as the two studies produced convergent findings [22, 23].

### 2.2 Stage 2: Development of Trigger Films

The scripts of the animated trigger films were based on key themes from the qualitative study. The themes included barriers and facilitators to physical activity in relation to capability, opportunity, and motivation [24]. In stage 2, we worked with a video producer to generate two trigger films. The first one provided different scenarios of four characters highlighting the challenges people experience when trying to conduct exercise, the second had scenarios featuring another four characters highlighting the support they have or hope to have to motivate their physical activity (Supporting Information: Material 2). These trigger films were used in stage 3 as part of the co‐design process.

### 2.3 Stage 3: Co‐Design Workshops

In this stage, a series of workshops was conducted following the inspiration and ideation phases of the Design Thinking method.

#### 2.3.1 Sample

The sample comprised people with young‐onset type 2 diabetes, their family members or friends, and healthcare professionals. Convenience sampling was employed to recruit participants from the Department of Endocrinology and Metabolism at the Third Affiliated Hospital, Sun Yat‐sen University in Guangzhou, China. Eligibility criteria for participants are listed below:

##### 2.3.1.1 People with Diabetes

People diagnosed with type 2 diabetes, aged between 18 and 40 years, of Chinese origin, living in mainland China, and fluent in Mandarin. Participants were ineligible if they lacked the capacity to consent.

##### 2.3.1.2 Family Members or Friends

People who provided informal support to people with young‐onset type 2 diabetes, including family members or friends.

##### 2.3.1.3 Healthcare Professionals

Healthcare professionals responsible for supporting people with young‐onset type 2 diabetes.

The research team distributed study leaflets to potential participants. Those interested received an information sheet with a consent form, with a minimum of 24 h to review before providing informed consent. Upon agreeing to participate, they were asked their preferred workshop(s) and given corresponding schedules. Participants had the option of attending multiple workshops. The sample size was determined based on the criteria proposed by Morgan [25], who recommended including a total of 3 to 5 groups, with each group comprising a minimum of 6 to 10 participants.

#### 2.3.2 Procedures

Data were collected using interactive workshops. Workshops 1–3 took place face‐to‐face at the hospital, and workshop 4 took place through a Tencent meeting. Activities included journey mapping, prioritisation exercises, co‐design mapping, and interactive software (Mentimeter). Two researchers facilitated each session: one leading the discussion, the other monitoring workshop fidelity and group dynamics. Workshops were audio recorded with consent. Reimbursements of ¥100 (£10.64) per hour were given to participants as a gesture of appreciation for their time.

##### 2.3.2.1 Workshop 1: Inspiration

Two parallel workshops were run, with one for individuals with diabetes and the other for healthcare professionals.

##### 2.3.2.2 Workshop 1.1: Engaging People With Diabetes and Gathering Experiences

The activities included a 30‐circle activity, trigger films, and journey mapping [14, 26]. After a brief introduction to the project, the 30‐circle activity served as a warm‐up session to encourage divergent design thinking. Each participant was given a 30‐circle worksheet and was asked to transform circles into recognisable objects related to physical activity. They were then invited to introduce the objects with their physical activity experience.

Participants then watched a trigger film on barriers to physical activity and were asked to consider their own challenges to physical activity, which they wrote onto post‐it notes and placed on a journey map poster illustrating different exercise phases (i.e., before exercise, during exercise, after exercise, other moments). Participants were invited to explain the challenges they have, and a representative summarised the main and common challenges. Then the second trigger film on facilitators to physical activity was shown. As before, participants were asked to write down what they found had helped them or might help them regarding physical activity on post‐it notes and place these on a journey map. Participants were invited to introduce their post‐it notes, and a representative summarised the main and potentially needed support strategies they would need.

##### 2.3.2.3 Workshop 1.2: Engaging Healthcare Professionals and Gathering Experiences

The activities in this workshop included viewing trigger films and journey mapping activities [27]. Following a brief introduction to the workshop, the healthcare professional group watched the second trigger film on facilitators to exercise. They were asked to write down the support that they could provide to people with young‐onset type 2 diabetes to further motivate their physical activity levels on post‐it notes and place these on a journey map. Participants were invited to share the ideas written on their post‐it notes, and a representative summarised the discussion. Then, the first trigger film was shown, and participants were asked to consider how to help the characters in the trigger film develop strategies for their exercise challenges. Workshop facilitators also brought challenges and ideas from workshop 1.1 to the healthcare professional group for discussion.

##### 2.3.2.4 Workshop 2: Joint Workshop With People With Young‐Onset Type 2 Diabetes, Their Family Members, and Healthcare Professionals

All participants from workshops 1.1 and 1.2 were invited to workshop 2, where the trigger films were screened again. Representatives presented their ideas and solutions to promote physical activity; participants then exchanged their perspectives. Finally, a prioritisation exercise was conducted, where participants rated a list of candidate intervention elements and BCTs on a 5‐point Likert scale, ranging from ‘not important’ to ‘very important’, to generate a list of priorities for intervention elements and BCTs.

##### 2.3.2.5 Workshop 3: Ideation ‐ Co‐Design Workshop

Workshop 3 was also an integrated workshop. The participants were asked to provide feedback on outputs from earlier workshops presented by the facilitator and then directed to position cards containing the intervention elements and BCTs on a prioritisation circle poster. The poster comprised three circles where elements could be placed: those in the central circle were deemed necessary, the first outer ring indicated that they were important but not necessary, the second outer ring signified being of less importance. Participants were divided into two groups and asked to prioritise intervention elements and BCTs. A representative for each group was selected to present their discussion, and opportunities were provided for other group members to share their thoughts.

The results from the prioritisation circle activity informed the discussion order of the intervention design, the co‐design map was then used to develop the intervention prototype (structured with 5 ‘W’ questions: what, who, when, where, how). The group considered how the intervention could be implemented, including the possible content, support, frequency, duration, location, and appropriate delivery formats.

##### 2.3.2.6 Workshop 4: Feedback and Celebration

The intervention prototype designed from workshops 3 was presented. Participants were invited to provide feedback on the name, utility, design, and content of the intervention components. Participants were also invited to complete an open‐ended survey to provide feedback on the workshops. This final workshop was also framed as a joint celebration of what had been achieved so far and allowed people to consider possible future directions for the intervention development.

### 2.4 Data Analysis

All qualitative data from the four workshops were transcribed verbatim and analysed thematically. Transcripts were reviewed for accuracy and translated into English by a researcher who was bilingual to enable analysis by English‐speaking co‐authors. The analysis followed Braun and Clarke's method for reflexive thematic analysis: 1. verbatim transcription in Mandarin for all discussions and translated into English; 2. familiarisation of the data by repeated reading of the outputs from the workshops, including transcripts, post‐it notes, and field notes; 3. generation of initial codes from the data inductively; 4. generation of common themes by comparing the relations between the ideas related to the prioritised interventions elements and BCTs; 5. reviewing themes; 6. defining and naming themes, the data were discussed with all authors [28].

To ensure rigour and trustworthiness of the study, we adhered to four criteria: credibility, dependability, confirmability, and transferability [29, 30]. Credibility was assured through peer debriefing, which involved regular discussions within the research team about the research process and data interpretation. Credibility was further supported through data triangulation, as data were collected from different populations, including patients, family members, and healthcare professionals. To ensure dependability and confirmability, workshop moderators kept reflexive notes, and participant quotes were included to support the identified themes and align the research team's interpretation with the data. Transferability was guaranteed by providing thick descriptions of the study context, participants, and the research processes, enabling readers to assess if the findings are transferable to other settings [29, 30].

The data from the prioritisation exercise in workshop 2 were used to rank the 5 items for intervention elements and 23 items for BCTs. Each item was scored from 1 (not important) to 5 (very important). Agreement was established according to the following inter‐rater reliability measures: a 70% consistency in scoring items as ‘important’ and ‘very important’; a mean value of 3.5; and an interquartile range ≤ 1 [31, 32, 33]. IBM SPSS 29 was used for quantitative estimation.

## 3 Results

Of 49 interested participants, 24 were excluded due to: ineligibility (*n* = 8), scheduling conflicts (*n* = 12), loss of interest (*n* = 3), and being unreachable (*n* = 1). Totally, 25 participants attended workshops: 12 people with type 2 diabetes (median age 31.5, interquartile range 7.25), 12 healthcare professionals (median age 33, interquartile range 8.75; 1 diabetes educator, 7 diabetes specialist nurses, 2 general nurses, 1 diabetes specialist physician, and 1 physiotherapist), and one family member. Half of the participants identified as women (*n* = 13; Table 1).

**Table 1:** Overview of the participants (*N* = 25).

Participants	*n* (%)	W 1.1 (*n* = 7)	W 1.2 (*n* = 10)	W 2 (*n* = 15)	W 3 (*n* = 9)	W 4 (*n* = 7)
**People with type 2 diabetes**	**12 (48)**	**7**		**4**	**3**	**4**
Age, years						
Median (IQR)	31.5 (7.25)					
Gender						
Male	10 (40)					
Diabetes duration, months						
Median (IQR)	11 (36.5)					
**HCPs**	**12 (48)**		**10**	**10**	**6**	**3**
Age, years						
Median (IQR)	33 (8.75)					
Gender						
Female	11 (44)					
**Family member, male**	**1 (4)**			**1**		
Age, years	33					
**Workshop duration, minutes**		**146**	**67**	**80**	**270**	**81**

Abbreviations: HCPs, health care professionals; IQR, interquartile range; W, workshop.

### 3.1 Results From Workshops

#### 3.1.1 Barriers and Facilitators to Physical Activity in People With Diabetes

After watching the trigger films, which were developed based on the qualitative study, participants reflected on their own experiences. Themes that generated in the workshops closely aligned with those identified in earlier research. The most common barriers to physical activity reported by participants included: lack of time, negative self‐efficacy, adverse weather conditions, and isolation. Other factors, such as the costs of exercise, negative emotions (e.g., depressive thoughts), and physical health status, were also mentioned.

If there are barriers, they might be what was mentioned earlier, sometimes, when going out, as that guy just said, whether it's travelling for work or being on a construction site, doing this kind of job means even mealtimes may not always be regular. In that case, there's not much that can be done. However, I'll find my own way; for example, if my lunchtime is irregular, I'll increase my exercise in the evening to make up for it.

(Individual with type 2 diabetes, S5)

Participants believed that to improve physical activity levels, developing greater self‐efficacy (self‐discipline/self‐awareness) was essential. The support most needed by participants was health information (preferably via apps or other eHealth platforms or from healthcare professionals) and emotional support from family members and friends to encourage them.

I feel that the people around me are quite supportive. For example, because I'm involved in exercise‐based diabetes management, my best friend always forwards weight‐loss videos to me whenever he sees them on TikTok. After the New Year, he set a goal for me: your goal this year is to lose weight, don't worry about anything else. The doctor told me very clearly that as long as I lose weight, I'll basically be fine. He even gave me a sort of guarantee and said it to me just like that.

(Individual with type 2 diabetes, S7)

Additionally, participants wanted peer support from others with diabetes for exercise companions, encouragement, and social interaction. Participants also reported that goal setting and self‐incentives would increase their motivation for physical activity.

#### 3.1.2 Healthcare Professionals’ Experience in Supporting Physical Activity in People With Diabetes

Healthcare professionals suggested strategies for promoting physical activity for people with young‐onset type 2 diabetes. These included: individualised exercise plans; goal setting (behaviour and outcome); improving knowledge of diabetes and physical activity; promoting social interaction via exercise clubs and online diabetes groups. Improving the accessibility of exercise equipment, providing social support (e.g., advising people with diabetes to exercise with family members or friends or having exercise instruction from a personal trainer), and providing emotional support were also suggested by healthcare professionals.

I think most people with diabetes know that exercise is important, but it's difficult to put into practice. They often say they're either too busy or have various other reasons. I'm not sure whether they're just making excuses or if there are other factors at play, but I believe the key is to create a personalised plan based on their schedule and interests, and to help them enjoy exercise.

(Diabetes specialist nurse, H4)

A key gap identified was the lack of eHealth resources for diabetes fitness in China. Participants suggested creating an online platform offering tailored exercise regimens, peer social interaction with peers, online consultations, exercise reminders, and encouragement.

My idea is to create an eHealth platform specifically for young people with type 2 diabetes. During exercise, it could provide motivational messages. After the workout, an encouraging video could be played. After a certain period, the doctor could offer a summary of progress and words of encouragement, as well as answer questions, either through a call or directly via a feature within the app.

(Diabetes specialist physician, H3)

To address the challenges to physical activity that those with young‐onset type 2 diabetes may have, health professionals stressed the importance of increasing people's self‐efficacy in relation to exercise and the need for individualised plans.

#### 3.1.3 Joint Discussion With All Stakeholders

There was agreement between those with diabetes and healthcare professionals on the challenges to physical activity. The healthcare professionals emphasised self‐efficacy and professional knowledge, and people with diabetes highlighted the need for professional guidance. The physiotherapist recommended flexibility in exercise, suggesting that it could be tailored to individual preferences:

When prescribing exercise for conditions like diabetes, it is typically moderate‐intensity and designed for the long term. Maintaining your heart rate within a specific target range is important. That said, adhering to such a prescription can be challenging for many people. However, the approach can be made flexible and tailored to individual preferences. For instance, some might enjoy square dancing, while others might prefer playing badminton, the type of activity can vary. I believe this approach would improve adherence for many people, as it allows for flexibility in achieving exercise goals.

(Physiotherapist, H12)

Almost all participants agreed on the benefit of developing an online platform for diabetes fitness to deliver potential intervention elements.

I think developing an eHealth platform would be quite beneficial. You could input your height, weight, blood lipid levels, age, and other relevant details, and it would generate an appropriate exercise plan for you. Additionally, it could provide information on how much your blood sugar levels might decrease and how many calories you would burn after completing the workout. That would be useful.

(Diabetes specialist nurse, H7)

#### 3.1.4 Prioritisation of Physical Activity Intervention Elements and Bcts

In the prioritisation exercise, guidance from professionals, physical activity knowledge, and self‐monitoring emerged as the most important intervention elements. Key BCTs included graded tasks, action planning, goal setting and review (behaviour and outcome), problem solving (Supporting Information: Material 1).

These findings were confirmed in the prioritisation circle activity in workshop 3. Professional guidance and physical activity knowledge were deemed necessary in both groups, followed by self‐monitoring, emotional support, and interactive physical activity. For the BCTs, goal setting/reviewing and action planning were deemed necessary, followed by graded tasks, instruction and demonstration of the behaviour, behavioural practice/rehearsal, and feedback (behaviour and outcome). Suggested activities aligned with BCTs included exercise goal and plan, engagement monitoring, health information, feedback mechanism, emotional support, social interaction, and behavioural comparison. The preferred delivery mode was online, with face‐to‐face options for behaviour feedback and enhancement. It was suggested that the intervention duration should be at least 3 months. Training in exercise and diabetes for facilitators was also suggested.

#### 3.1.5 Intervention Prototype

The prototype intervention ‘IPAYD’ (Improving Physical Activity for people with Young‐onset type 2 Diabetes) was presented to participants. The final conceptual model incorporated an online platform, one‐to‐one consultations, and optional group sessions. The platform featured individualised goal setting and planning, exercise monitoring, educational resources, and a peer support forum. An AI‐driven chat function was proposed for use in exercise goals to enhance engagement. Exercise goals are developed following American College of Sports Medicine (ACSM) guidelines [34], considering pre‐exercise risk assessments, previous exercise behaviour, baseline characteristics, user preferences, and perceived barriers to exercise.

Participants reviewed the platform's design and functionality and raised privacy concerns about the use of personal data in exercise ranking, which could be addressed by allowing users to opt in or out. Additional suggestions included using vibrant colours to match different exercise topics, adding resources on medication and diet, and enabling automatic syncing of previously entered data to reduce user burden. The participants agreed that one‐to‐one sessions would be necessary for engagement and for professional feedback related to their exercise plan, but having the option to do this virtually would be useful. Group sessions were viewed as important in providing social support.

### 3.2 Feedback After Workshops

Anonymous feedback after each workshop was generally positive. Suggestions were incorporated wherever possible in subsequent workshops (e.g., scheduling sessions outside working hours and sharing agendas in advance). Participants felt listened to and appreciated the opportunity to engage with other stakeholders. Healthcare professionals appreciated the well‐conducted workshops and the co‐design approach, which is novel in China. Participants considered the hospital setting to be an appropriate venue for the workshops.

### 3.3 Logic Model

**Figure 2:**
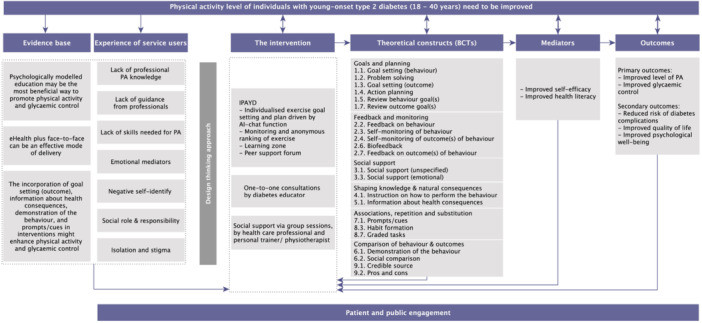
Logic model of the IPAYD intervention. IPADY, improving physical activity for people with young‐onset type 2 diabetes.

A logic model of showing the structure, mechanisms, and outcomes of the IPAYD is shown in Figure 2. This model incorporates the outputs from all stages of the study, including evidence and theory derived from the early stages, and outputs from subsequent co‐design workshops. This intervention is unique in that it was tailored to the context of Chinese people with young‐onset type 2 diabetes, developed through a rigorous co‐design process. Additionally, it incorporates eHealth and BCTs, adheres to ACMS's guidelines for individualised exercise goal setting and planning [34]. We plan to establish the feasibility and acceptability of the developed intervention before assessing its effectiveness and cost‐effectiveness in a randomised controlled trial.

## 4 Discussion

This paper presents the development of a multimodal intervention to support people with young‐onset type 2 diabetes to increase their physical activity levels. The co‐design approach generated and prioritised the intervention content, delivery, and underpinning behaviour change theory, reflecting the context of Chinese people with young‐onset type 2 diabetes. This is particularly important given that there is no such intervention available specifically for this population.

The proposed logic model for the intervention has an emphasis on eHealth technologies. This approach may better fit the lives of younger adults with type 2 diabetes who have work and family responsibilities and have familiarity with online technology [35]. eHealth technologies (e.g., website, smartphone‐based apps, wearable devices, and AI) have started to play a significant role in people's daily lives and are increasingly used in healthcare and sports medicine [36]. Such technology improves user accessibility and flexibility and is particularly beneficial for people in remote or underserved areas. The participants in this study identified deficits in their knowledge of exercise and its potential benefits, and felt this could be addressed with online/mobile education. It has been shown that providing targeted information can increase self‐efficacy and motivation [37]. Empirical studies, however, note that not all people with diabetes have equal access to health information associated with their condition, and that resources do not represent all members of society equitably [38]. While younger populations may be more likely to engage with online technology, the participants in this study preferred a blended approach with face‐to‐face interactions alongside digital materials in the intervention. Existing evidence also suggests that blended learning can be effective in improving glycaemic control and physical activity levels in people with diabetes [36].

The co‐design process also identified BCTs that could be embedded into the intervention to increase people's motivation and self‐efficacy, including using goal setting and harnessing social support. Using psychological theory to support behaviour change interventions is important and has been shown to enhance outcomes [39, 40]. While individualised goal setting is not new in physical activity interventions [40], it has not been considered in physical activity interventions specifically for the young‐onset type 2 diabetes population, with current exercise guidelines for this group adopted from those for old people with diabetes [7]. Individualised goal setting is personally tailored and thus allows for flexibility and adaptability, making goals more relevant and achievable [10, 39]. Social support from peers was also identified as important in providing emotional, instrumental, and informational support through shared experiences and learning [41]. Other BCTs incorporated into the intervention were problem solving, demonstration of the behaviour, and social comparison. These BCTs were included to facilitate observational learning, psychological empowerment, intrinsic motivation, and increase physical activity engagement [39]. Hence, the intervention framework developed in this study reflects the needs of younger adults with diabetes and incorporates a number of complementary elements, delivery mechanisms, and underpinning theories to support behaviour change in relation to physical activity in this population.

### 4.1 Strengths and Limitations

The key strength was the use of a co‐design approach, which ensured the relevance of the intervention for the target population. A further strength of this study was that participants themselves considered optimal delivery methods and appropriate BCTs [39]. We adhered to core principles of co‐design, with conscious efforts to manage power dynamics and promote equitable participation [14, 27]. Strategies included: workshop moderators were trained in participatory methods, they encouraged input from all participants to prevent dominance by any individual; employing reflective practice and maintaining openness to criticism; valuing both lived experience and professional expertise throughout the workshops.

Several limitations should be acknowledged. While co‐design requires equal collaboration among healthcare professionals and people with diabetes, divergences emerged between lived experience perspectives and views from healthcare professionals, which are contextualised in the structural and cultural conditions present in the healthcare system. Equally, power dynamics in terms of hierarchies between young people and health professionals were likely to have been in play at points during the joint workshops. Indeed, some participants might have withheld crucial information or provided socially desirable responses due to apprehension about speaking in a group setting or in front of clinical experts. The research team consciously managed these dynamics as much as possible to foster equitable participation. Encouragingly, there was clear evidence of a convergence of views about the intervention model in the final workshop.

The use of convenience sampling may have limited the transferability of our findings due to selection bias. Gender imbalance in healthcare professionals and limited family involvement further constrained representativeness. These can be explained by practical and contextual factors, including the cost and time efficiency of the sampling, accessibility of the targeted participants, the predominance of women in the diabetes care workforce, and the competing family and employment responsibilities of potential family participants. In addition, the ratio of patients to health care professionals in workshops 2 and 3 created a potential authority imbalance that might constrain the patients’ voice. It may also be the case that people who agreed to take part in the study had different views about exercise than those who did not attend. This study was only done in one site in China; it will need to be tailored for other countries or regions. Nonetheless, these limitations were mitigated by achieving an overall gender balance in participants, overall numerical balance between patient and health care professionals (12:12), and including individuals from different age groups and clinical backgrounds. Future research should consider multi‐site recruitment to capture greater geographical, socioeconomic, and cultural diversity, ensure better gender balance through purposive or stratified sampling, and explore strategies to enhance family engagement. Additionally, whilst participants were enthusiastic about the intervention, concerns were raised about cost, attendance, facilitator suitability, and the authenticity of self‐monitoring data. Such issues should be considered in future feasibility studies.

## 5 Conclusions

This study has developed a novel, co‐designed intervention to promote physical activity in people with young‐onset type 2 diabetes, a population for whom tailored interventions are scarce. The intervention was shaped by their needs and preferences, identifying key components, delivery mechanisms, and BCTs to ensure feasibility and acceptability.

## Author Contributions

**Xiaoyan Zhao:** conceptualisation, methodology, investigation, formal analysis, funding acquisition, data curation, project administration, writing – original draft, writing – review and editing. **Angus Forbes:** conceptualisation, methodology, data curation, project administration, supervision, writing – review and editing. **Haya Abu Ghazaleh:** conceptualisation, methodology, data curation, project administration, supervision, writing – review and editing. **Li Cheng:** methodology, writing – review and editing. **Xiaodi Guo:** methodology, writing – review and editing. **Maria Duaso:** conceptualisation, methodology, data curation, project administration, supervision, writing – review and editing.

## Ethics Statement

Ethical approvals were obtained from the ethics committees at King's College London (MRSP‐23/24‐41884), and the Third Affiliated Hospital, Sun Yat‐sen University (II2024‐165‐01).

## Conflicts of Interest

The authors report there are no competing interests to declare.

## Supporting information

Supplementary material.

## Data Availability

The data supporting the findings of this study are available from the corresponding author upon reasonable request.
