# Supplementary material for: Co‐Designing a Multimodal Physical Activity Intervention for Individuals With Young‐Onset Type 2 Diabetes (18–40 Years) in China
Source: Health Expect. 2026 Feb 3;29(1):e70580. doi: 10.1111/hex.70580 (PMC12868930; doi:10.1111/hex.70580)
Supplement: Supplementary file 1 — Supplementary material. [file HEX-29-e70580-s001.docx]

**Supplementary material 1** Results from prioritisation exercise (*N=15*)

| **Candidate intervention elements and BCTs** | **PA^&^** | **IQR^#^** | **Mean (SD)^*^** |
| --- | --- | --- | --- |
| ***Intervention elements*** |  |  |  |
| 1. Guidance from professionals (individualised exercise guidance) | 93.7 | 1 (4-5) | 4.60 (0.632) |
| 1. Physical activity knowledge | 93.7 | 1 (4-5) | 4.60 (0.632) |
| 1. Self-monitoring | 93.7 | 0.5 (4.5-5) | 4.53 (1.060) |
| 1. Interactive physical activity | 80 | 1 (4-5) | 4.20 (0.775) |
| 1. Emotional support | 80 | 1 (4-5) | 4.13 (1.407) |
| ***BCTs*** |  |  |  |
| 1. Goal setting (behaviour) | 80 | 1 (4-5) | 4.40 (0.828) |
| 1. Problem solving | 93.7 | 1 (4-5) | 4.40 (0.632) |
| 1. Goal setting (outcome) | 93.7 | 1 (4-5) | 4.40 (0.632) |
| 1. Action planning | 93.7 | 1 (4-5) | 4.43 (0.622) |
| 1. Review behaviour goal(s) | 86.7 | 1 (4-5) | 4.20 (0.676) |
| 1. Review outcome goal(s) | 93.7 | 1 (4-5) | 4.33 (0.617) |
| 1. Monitoring of behaviour by others without feedback | 66.7 | 1 (3-5) | 3.73 (1.335) |
| 1. Feedback on behaviour | 80 | 1 (4-5) | 4.27 (1.163) |
| 1. Self-monitoring of behaviour | 93.7 | 1 (4-5) | 4.42 (0.623) |
| 1. Monitoring of outcome(s) of behaviour without feedback | 80 | 1 (4-5) | 4.13 (1.125) |
| 1. Feedback on outcome(s) of behaviour | 80 | 1 (4-5) | 4.40 (0.828) |
| 1. Social support (unspecified) | 80 | 1 (4-5) | 4.00 (1.069) |
| 1. Social support (practical) | 66.7 | 1 (3-5) | 3.73 (1.335) |
| 1. Instruction on how to perform the behaviour | 53.3 | 1 (3-4) | 3.47 (1.246) |
| 1. Information about health consequences | 66.7 | 1 (3-5) | 3.93 (0.961) |
| 1. Demonstration of the behaviour | 80 | 1 (4-5) | 4.40 (0.828) |
| 1. Prompts/cues | 73.3 | 1.5 (3.5-5) | 4.20 (0.862) |
| 1. Behavioural practice/rehearsal | 80 | 1 (4-5) | 4.07 (0.884) |
| 1. Graded tasks | 100 | 1 (4-5) | 4.47 (0.516) |
| 1. Credible source | 80 | 1 (4-5) | 4.33 (0.816) |
| **Supplementary material 1** (continued) |  |  |  |
| 1. Pros and cons | 86.7 | 1 (4-5) | 4.33 (0.724) |
| 1. Restructuring the physical environment | 73.3 | 1 (3.5-4.5) | 3.73 (1.280) |
| 1. Adding objects to the environment | 73.3 | 1 (3.5-4.5) | 3.73 (1.280) |

**Notes:** 5-point Likert scale from 1 = not important to 5 = very important. BCT: behaviour change technique; ^&^PA: percentage of agreement, a sum score of those who ticked the last two responses (important, very important), the agreement level is set as 70%; ^#^IQR (interquartile range) value equal to or lower than 1 in a five-point Likert scale as consensus level; ^*^The mean of the responses is a common measure, and the agreement threshold was set as 3.5.

**Supplementary material 2**

- Trigger film 1 - Barriers to physical activity experienced by people with young-onset type 2 diabetes, held at [https://doi.org/10.18742/28382225](https://eur03.safelinks.protection.outlook.com/?url=https%3A%2F%2Fdoi.org%2F10.18742%2F28382225&data=05%7C02%7Cxiaoyan.zhao%40kcl.ac.uk%7C117ef2cf41e845aa9f2008dd50fdfa20%7C8370cf1416f34c16b83c724071654356%7C0%7C0%7C638755773931524709%7CUnknown%7CTWFpbGZsb3d8eyJFbXB0eU1hcGkiOnRydWUsIlYiOiIwLjAuMDAwMCIsIlAiOiJXaW4zMiIsIkFOIjoiTWFpbCIsIldUIjoyfQ%3D%3D%7C0%7C%7C%7C&sdata=NX%2BbIOkf7q3R7uRolS%2B0shZHG4PFFttAFcOOuXK2RmQ%3D&reserved=0)
- Trigger film 2 - Facilitators to physical activity experienced by people with young-onset type 2 diabetes, held at [https://doi.org/10.18742/28382282](https://eur03.safelinks.protection.outlook.com/?url=https%3A%2F%2Fdoi.org%2F10.18742%2F28382282&data=05%7C02%7Cxiaoyan.zhao%40kcl.ac.uk%7C117ef2cf41e845aa9f2008dd50fdfa20%7C8370cf1416f34c16b83c724071654356%7C0%7C0%7C638755773931546225%7CUnknown%7CTWFpbGZsb3d8eyJFbXB0eU1hcGkiOnRydWUsIlYiOiIwLjAuMDAwMCIsIlAiOiJXaW4zMiIsIkFOIjoiTWFpbCIsIldUIjoyfQ%3D%3D%7C0%7C%7C%7C&sdata=fcg88YfyS7qNPaGRUP3vUW5SWqt%2FSCBFh7%2BvFNa5sl4%3D&reserved=0)
